# Metabolic and Phenotypic Changes Induced during N-Acetylglucosamine Signalling in the Fungal Pathogen *Candida albicans*

**DOI:** 10.3390/biomedicines11071997

**Published:** 2023-07-14

**Authors:** Somnath Sahoo, Sarika Sharma, Mahendra P. Singh, Sandeep K. Singh, Emanuel Vamanu, Kongara Hanumantha Rao

**Affiliations:** 1 Department of Biochemistry, School of Bioengineering and Biosciences, Lovely Professional University, Phagwara 144411, India; somnath93sahoo@gmail.com; 2 Department of Sponsored Research, Division of Research & Development, Lovely Professional University, Phagwara 144411, India; 3 Department of Zoology and Centre of Genomics and Bioinformatics, DDU Gorakhpur University, Gorakhpur 273009, India; mprataps01@gmail.com; 4 Indian Scientific Education and Technology Foundation, Lucknow 226002, India; 5 Faculty of Biotechnology, University of Agricultural Sciences and Veterinary Medicine, 011464 Bucharest, Romania; 6 Department of Biochemistry/Bioinformatics, School of Sciences, Gandhi Institute of Technology and Management (GITAM Deemed to be University), Visakhapatnam 530045, India

**Keywords:** *Candida albicans*, morphogenetic, N-acetylglucosamine, metabolites, GC–MS, FE-SEM, amphotericin B

## Abstract

The human commensal yeast *Candida albicans* is pathogenic and results in a variety of mucosal and deep tissue problems when the host is immunocompromised. Candida exhibits enormous metabolic flexibility and dynamic morphogenetic transition to survive under host niche environmental conditions and to cause virulence. The amino sugar N-acetylglucosamine (GlcNAc) available at the host infection sites, apart from acting as an extremely good carbon and nitrogen source, also induces cellular signalling in this pathogen. In *C. albicans*, GlcNAc performs multifaceted roles, including GlcNAc scavenging, GlcNAc import and metabolism, morphogenetic transition (yeast—hyphae and white—opaque switch), GlcNAc-induced cell death (GICD), and virulence. Understanding the molecular mechanism(s) involved in GlcNAc-induced cellular processes has become the main focus of many studies. In the current study, we focused on GlcNAc-induced metabolic changes associated with phenotypic changes. Here, we employed gas chromatography–mass spectrometry (GC–MS), which is a high-throughput and sensitive technology, to unveil global metabolomic changes that occur in GlcNAc vs. glucose grown conditions in Candida cells. The morphogenetic transition associated with metabolic changes was analysed by high-resolution field emission scanning electron microscopy (FE-SEM). Metabolite analysis revealed the upregulation of metabolites involved in the glyoxylate pathway, oxidative metabolism, and fatty acid catabolism to probably augment the synthesis of GlcNAc-induced hypha-specific materials. Furthermore, GlcNAc-grown cells showed slightly more sensitivity to amphotericin B treatment. These results all together provide new insights into the development of antifungal therapeutics for the control of candidiasis in humans.

## 1. Introduction

An opportunistic human fungal pathogen such as *Candida albicans* causes candidiasis. It generally lives as a commensal on the mucosal sites of the mammalian gastrointestinal system and other body organs. However, in immunocompromised individuals and in people continuously undergoing antifungal treatment, Candida can cause a few mucosal and deep tissue disorders. The fourth most frequent nosocomial infection, more than half of which can be deadly, is caused by Candida [1]. At least 70% of healthy people have commensal *C. albicans* in their mouth, skin, female reproductive tract, and gastrointestinal tract [2,3,4,5,6]. *C. albicans* morphological flexibility is considered a crucial virulence factor. *C. albicans* can transform reversibly among yeast, pseudohyphal, and hyphal growth forms, which are important for establishing infection in the host [7,8]. Recently, there has been an increasing emphasis on understanding the connections of these various morphotypes with various host habitats and proclivity for virulence vs. commensalism. Various studies have established that exogenous stimulus like N-acetylglucosamine (GlcNAc), serum, or neutral pH effectively promote hyphal development [9,10,11]. This adaptability may make it easier for *C. albicans* to cope with different host environments while infected.

*C. albicans* exhibits enormous metabolic flexibility to utilize various carbon sources available at infection sites, which has been shown to be crucial for its survival, host niche colonization, and virulence [12,13,14]. The amino sugar N-acetylglucosamine (GlcNAc), present at infection sites of *C. albicans*, not only acts as a good carbon and nitrogen source but also an effective inducer of signalling to stimulate the expression of its own catabolic genes [15,16,17,18] and morphogenetic transitions [19,20,21,22]. Previous studies have also indicated that disruption of the GlcNAc utilization pathway leads to attenuated virulence in mouse model candidacies. GlcNAc catabolism has also been shown to be important for colonization and establishing virulence inside the host, in several pathogens, including *Vibrio cholerae* [23], *Magnaporthe oryzae* [24], and *Leishmania donovani* [25]. Nevertheless, the functional significance of several metabolic enzymes involved in the formation of metabolic intermediates remains elusive. Only a few recent studies have examined the probable role of novel metabolic enzymes involved in GlcNAc metabolism [12,18]. By connecting to the GlcNAc sensor (Ngs1) through the GlcNAc-specific transporter (Ngt1), internalized GlcNAc can directly activate signalling. GlcNAc is converted to GlcNAc-6-P by GlcNAc kinase (Hxk1), which is then further catabolized by sequential deacetylase (Dac1) to create Glucosamine-6-p and deaminase (Nag1) to generate glucose-6-phosphate, which is used as an energy source, or turned into UDP-GlcNAc, which is linked to anabolic processes. GlcNAc-6-P produced internally is utilized in anabolic processes such as chitin formation, GPI anchoring, and N-glycosylation but is not a component of the GlcNAc signalling system. GlcN-6-P synthase (Gfa1) and GlcNAc-6-P acetyltransferase (Gna1) work together in tandem to create GlcNAc-6-P from fructose-6-P during glycolysis. GlcNAc-6-P is converted to UDP-GlcNAc (Uap1) by the activities of phosphor acetylglucosamine mutase (Agm1) and UDP GlcNAc pyrophosphorylase [17].

Metabolic flexibility has been shown to provide immune defence, antifungal resistance, virulence, and several other physiological advantages to *Candida* [26]. Amphotericin B (AmpB) is widely used as an antifungal drug in humans. Despite its side effects of causing severe nephrotoxicity, it has become the choice for treating fatal systemic fungal infections such as candidiasis. The side effects are mitigated by designing novel AmpB drug formulations (liposomes, emulsions, and nanoparticles) that aim to lower the amount of free AmpB in the blood to levels below the threshold of toxicity and ensure that the drug is delivered to the site of need as quickly as feasible. The biological activity of AmpB is thought to be the formation of transmembrane holes capable of acting as ion channels impeding cell electrostasis [27]. It has also been shown to bind to the cell walls of mature cells and thereby prevent budding-stage daughter cells from forming a functional cell wall [28]. Understanding the sensitivity of *C. albicans* against AmpB treatment in response to growth on various alternative carbon sources, such as GlcNAc, amino acids, and non-fermentative carbon sources apart from glucose, plays an important role in developing novel formulations.

Given the significance of GlcNAc utilization and its metabolism in *C. albicans*, there is an immense need to study and analyse the metabolic pathways that operate during GlcNAc utilization. In this study, we focused on the analysis of differentially upregulated metabolites in GlcNAc-grown cells compared with glucose-grown cells. Metabolite analysis by using gas chromatography–mass spectrometry (GC–MS) revealed the presence of metabolites such as ergosterol, farnesol, and alanine in response to GlcNAc. GlcNAc-grown cells showed more sensitivity to the antifungal compound amphotericin B than glucose-grown cells. The yeast and hyphal morphogenetic transition states triggered in response to glucose and GlcNAc were examined with FE-SEM.

## 2. Materials and Methods

### 2.1. Media and Culture Conditions

A 15% glycerol frozen stock of *C. albicans* (SC5314) was kept at −80 °C for further use. A sufficient culture amount was taken and streaked on YPD agar plates (2% dextrose, 2% Bacto peptone, 1% yeast extract, 2% Bacto Agar) (HiMedia Laboratories Pvt. Ltd, Secunderabad, Telangana, India) and kept at 30 °C in incubator for 2 days for the growth of the fungal cells and then proceeded for general laboratory work. To revive the culture, fungal cells were newly streaked on new YPD agar petri plates every week, and the work was continued. Whether the culture was contaminated or not, the cells were checked under a microscope. Overnight cultures were grown in liquid YPD (HiMedia Laboratories Pvt. Ltd., Secunderabad, Telangana, India) media containing (2% dextrose, 2% Bacto peptone, 1% yeast extract) [29] at 75 rpm at 30 °C. For alternative carbon sources, we also prepared 5 mM (mM) GlcNAc and 2% glucose solution (Loba Chemie Pvt Ltd., Mumbai, Maharashtra, India) [30]. These solutions were sterilized with a 0.45 µm filter. Generally, the cells were grown in yeast nitrate base (YNB) media (HiMedia Laboratories Pvt. Ltd., Secunderabad, Telangana, India) with these carbon sources.

### 2.2. Field Emission-Scanning Electron Microscopy (Fe-SEM) to Check Morphology

**Figure 1:**
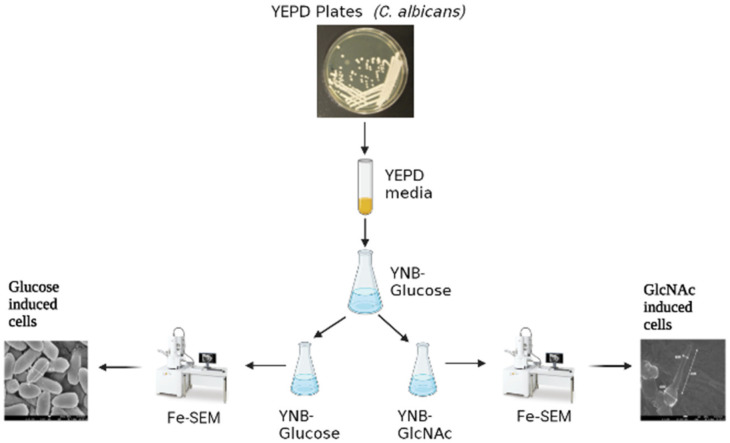
Methodology for Fe-SEM. YEPD/YPD: yeast, peptone, dextrose media; YNB-glucose/GlcNAC: yeast nitrogen base supplemented with either GlcNAc or glucose; Fe-SEM: field emission scanning electron microscopy.

First, glucose- and GlcNAc-induced cells were grown in YNB medium at 30 °C for 3 h. *Candida albicans* cells were collected by centrifugation at 8000 rpm. The cells were then fixed in 2.5% glutaraldehyde (Loba Chemie Pvt Ltd, Mumbai, Maharashtra, India) for two hours in 0.1 M-NaPO_4_ buffer, postfixed in buffered 1% osmium tetroxide (OsO4) (Loba Chemie Pvt. Ltd., Mumbai, Maharashtra, India) for two hours, and then washed with phosphate buffer PBS two to three times. Following ethanol gradient elution to dry the material, the following concentrations of ethanol were added: 30%, 50%, 70%, 85%, and 95%, each once (10 min/time), and 100% twice (20 min/time) [31]. The final steps included critical point drying, gold sputter coating, and FE-SEM viewing of the specimens (Figure 1).

An enhanced technique for capturing the microstructure picture of the materials or specimen is FE-SEM. Considering that gas molecules have the propensity to influence the electron beam and the produced secondary and backscattered electrons utilized for imaging, normal FE-SEM operations take place in a high vacuum. Zeiss Crossbeam 340 was utilized to take the microstructure picture and analyse the specimens in this investigation. Prior to the morphological examination, the specimens were taken within a vacuum drying room, and the samples were dried. After drying, the sample temperature was raised to room temperature. Poured into the culture after being taken off the heat; the coverslip is covered with carbonaceous conductive adhesive tape that is viscous on one side and light on the other and coated with gold. While morphology is being examined, parameters can be changed and organized.

### 2.3. High-Performance Liquid Chromatography (HPLC) to Check Any Difference in Induced Cells

High-performance liquid chromatography is generally known as HPLC. Chromatography is a separation technique, a chromatogram is the end product, and a chromatograph is an equipment used to carry out chromatography. Some of the essential elements of chromatographs are high-performance pumps for supplying solvent at a steady flow rate and columns, which are special equipment made for molecular separation. Only compounds that have been dissolved in solvents may be evaluated using HPLC. To analyse the elements and concentrations of each element in the sample, both qualitatively and quantitatively.

A 1%, *C. albicans* inoculum was taken from YPD plates and grown in YPD medium overnight for induction. Once more, 1% of this culture was added to the YNB-glucose medium for 12 h to promote growth. After checking the OD, the sample was centrifuged at 25 °C and 6000 rpm for 15 min, and the pellet was washed with autoclaved water. Finally, the cells were cultured in YNB-glucose and YNB-GlcNAc for 3 h and centrifuged at 4 °C and 8000 rpm for 10 min. Pellets were dipped in liquid nitrogen, ground with a mortar and pestle, homogenized in methanol–water in a 3:1 ratio, and vortexed with glass beads for 1 min, followed by ice treatment for a min [32]. This process was performed three times to lyse the cell membrane. Then, the cells were incubated for 1 h and centrifuged to obtain metabolites. These fractions were assayed for differences in proteins and metabolites by HPLC (HPLC-150722-70_160722_PDA_SS.lcm) at Lovely Professional University, Jalandhar, India with a PDA detector in the 2022_HPLC$July 2022 project.

### 2.4. Gas Chromatography and Mass Spectrometry (GC–MS) to Check the Inducible Metabolites

To identify different substances in a test sample, an analytical method known as gas chromatography–mass spectrometry (GC–MS) combines the benefits of gas chromatography and mass spectrometry. Moreover, traces of components may be discovered in objects that were previously thought to have broken down beyond recognition. It enables the examination and identification of even minute quantities of a chemical, similar to liquid chromatography–mass spectrometry. The term “GC–MS” refers to a technique that is used to conduct a 100% specific test that positively identifies the presence of a specific chemical and is known as the “gold standard” for forensic substance identification [33].

*C. albicans* was grown overnight in YPD medium from YPD plates. Then, 1% preculture was cultivated for 12 h on YNB-glucose medium. The pellet was washed with autoclaved water after centrifuging at 25 °C for 15 min at 6000 rpm after testing the OD. It was then centrifuged at 4 °C and 8000 rpm for 10 min after being cultured in YNB-glucose and YNB-GlcNAc for 3 h. We have followed the same procedure that has been standardized previously in HPLC for induced samples. Then, the sample was dried in a vacuum hot oven at 40 °C for 3 h, and a sample volume of 1 µL was obtained using a hot needle technique for GC–MS analysis [32].

### 2.5. Minimum Inhibitory Concentration (MIC) Using a Microplate Reader

Using the standard broth microdilution approach based on the recommendations of the Clinical Laboratory Standards Institute, the impact of AmpB on the development of *C. albicans*-induced cells was investigated previously, and we followed the same procedure [34]. In untreated 96-well polystyrene plates, AmpB (Sisco Research Laboratories Pvt. Ltd., Mumbai, Maharashtra, India) doses ranging from 0.1 to 100 g mL^−1^ were synthesized in YNB-medium. Control wells were those lacking the test chemicals. To achieve 1 × 10^3^ cells mL^−1^, 100 microliters of inoculum was mixed with 100 microliters of YNB-medium in each well. For 48 h, the plates were incubated at 35 °C. Using a microplate reader (Labtronics Model LT-1260, LPU, Jalandhar, India), the absorbance at 620 nm was measured to evaluate the growth. The minimum inhibitory concentration (MIC) for *C. albicans* growth was defined as the AmpB concentration where there was a 50% drop in absorbance in comparison to the control [35].

## 3. Results and Discussion

### 3.1. N-Acetylglucosamine Acts as the Sole Carbon Source for C. albicans

**Figure 2:**
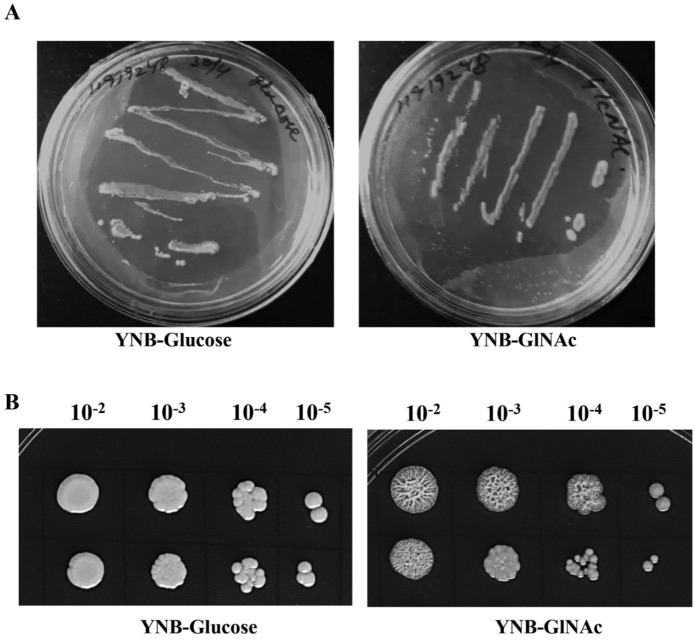
*Candida albicans* (SC5314) grown on YNB–glucose and YNB–GlcNAc media. (**A**) Growth pattern of the Candida strain streaked on YNB–glucose or YNB–GlcNAc plates and grown for 3 days at 30 °C. (**B**) Spot representing the growth of the Candida strain grown similarly to panel A for 2 days. GlcNAc–grown cells are elongated and form a smear colony, whereas glucose-induced cells are circular and distinct in the colony.

To comprehensively understand the significance of GlcNAc as a carbon source to promote growth and to induce morphogenetic transition in *C. albicans* (strain SC5314), we observed how these cells grew on YNB medium plates with GlcNAc while using glucose as a control by following the same method as in Tao, L. et al., 2017 [11]. We found that the strain is able to use GlcNAc as a sole carbon source, and cells are elongated and form a filamentous colony, whereas glucose-induced cells are circular and form smooth colonies, as shown in Figure 2.

### 3.2. Morphogenetic Changes in N-Acetylglucosamine-Induced Candida Cells

**Figure 3:**
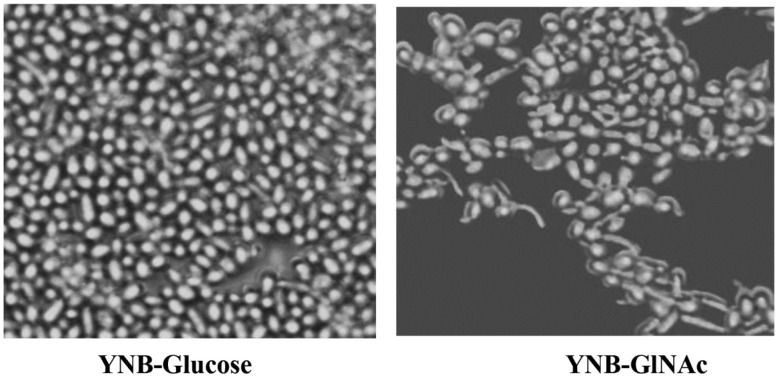
Microscopic image (40×) of *Candida albicans* (SC5314) grown on YNB-glucose and YNB-GlcNAc media. We can clearly define that in GlcNAc media, the cells form hyphae and are elongated in shape, whereas the cells grown in glucose media are circular in form.

Changes in the carbon supply alone can cause *C. albicans* to develop hyphae. Growing in glucose medium promotes the development of yeast and the substitution of GlcNAc promotes the growth of mycelium, as shown in Figure 3. The yeast cells of *C. albicans* are relatively elongated. Only after the germ tubes have grown to be rather long—a characteristic acquired after a few hours of development in GlcNAc-containing medium—distinct separation from mycelial cells can be determined. As previously mentioned in [36], after some time of incubation, the number of mycelial cells in the YNB-GlcNAc medium began to decline. This reduction was caused by the active reproduction of yeasts that had initially not reacted to the morphogenetic stimuli rather than the elimination of mycelial cells or the conversion of mycelia to yeast. Every time the impact of environmental factors on dimorphism is investigated, this result raises questions. The best outcomes were obtained when starvation and growth on GlcNAc were combined. Ninety percent of the cell population grew into hyphae after germination, and this percentage persisted for 24 h. Extremely long and branching filaments developed after an additional 48 h of incubation, but there was no increase in the number of yeast cells. Since they are unable to bud, this finding implies that the remaining yeast cells are dead.

### 3.3. Field Emission-Scanning Electron Microscopy Study

**Figure 4:**
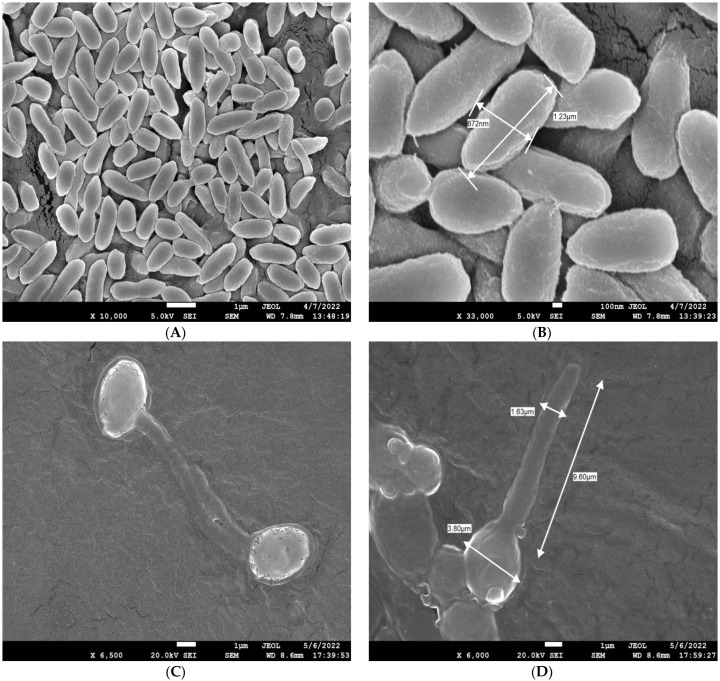
FE–SEM images of glucose– and GlcNAc–induced cells. (**A**) Images of glucose-induced cells seen through SEM at 10,000×; (**B**) glucose-induced cells at 33,000×; and (**C**,**D**) GlcNAc-induced cells at 6500× and 6000×, respectively, seen through SEM. Normal glucose-induced cells were 1.23 µm × 0.67 µm in size, whereas GlcNAc-induced cells were 9.60 µm × 3.80 µm in size. We can clearly differentiate between yeast and hyphal forms of *Candida albicans*.

Comparing the GlcNAc-treated cells to the glucose-grown control cells revealed observable structural alterations under the light microscope (Figure 3). Before proceeding with differential metabolomics analysis, we wanted to perform ultrastructural analysis of both GlcNAc- and glucose-grown cells. For this purpose, we used field emission scanning electron microscopy (FE-SEM, Joel) following the procedure described in Section 2. Compared to the glucose control, the GlcNAc-treated cells showed hyphal development. We can properly differentiate between them, and according to the report, the normal glucose-induced cells are 1.23 µm × 0.67 µm in size, whereas the GlcNAc-induced cells are 9.60 µm × 3.80 µm in size (Figure 4).

### 3.4. Metabolite Profiling

**Figure 5:**
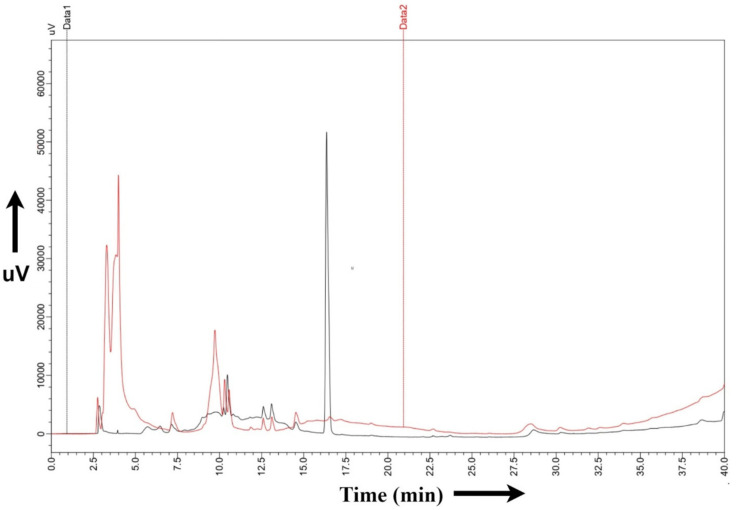
HPLC results of glucose- and GlcNAc-induced *Candida albicans* cells. Data 2 (red) represent peaks of different metabolites expressed in glucose-induced cells, whereas Data 1 (black) represent peaks of metabolites expressed in N-acetylglucosamine (GlcNAc)-induced cells. We can clearly see the difference in peaks in the two different carbon utilization processes. The main differences seen are in time periods 3.5 min and 16 min. It is the overlay plot of both glucose- and GlcNAc-induced cells.

**Figure 6:**
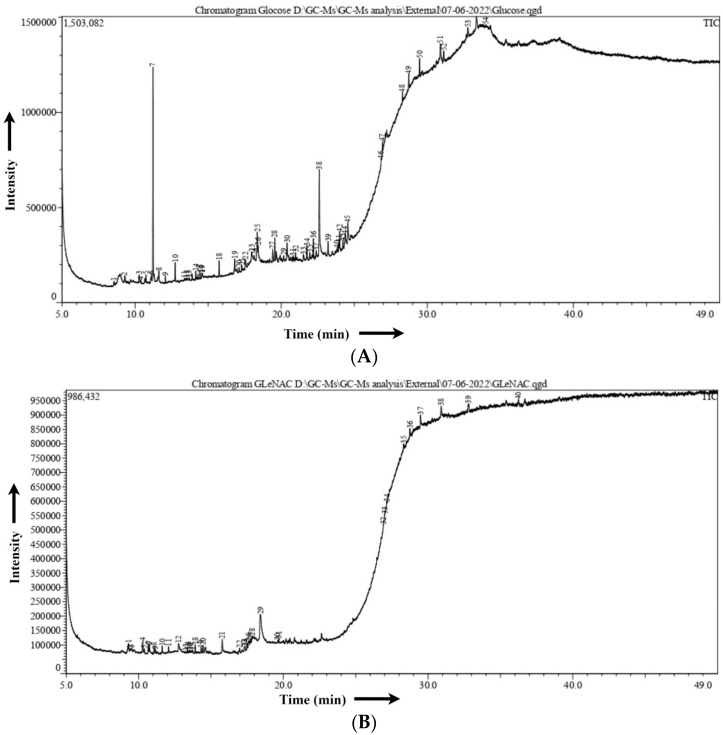
Gas chromatography–mass spectrometry analysis. (**A**) GC–MS result of glucose-induced cells. (**B**) GC–MS result of N-acetylglucosamine (GlcNAc)-induced cells. The intensity of the peaks of metabolites with respect to time was analysed and is explained later in table form.

**Figure 7:**
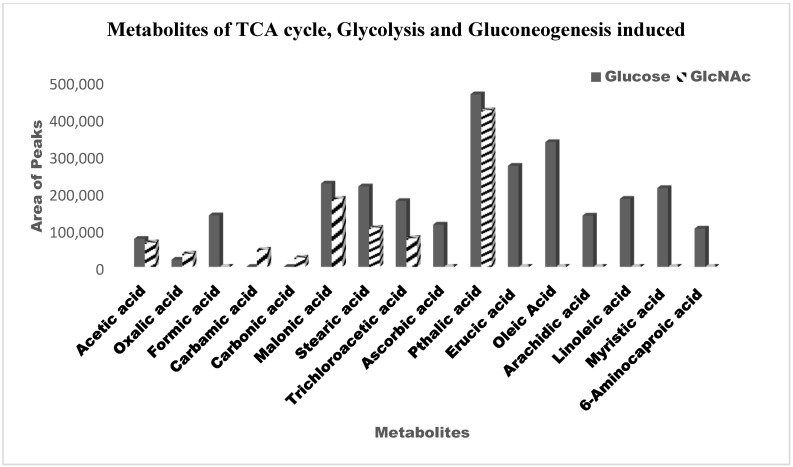

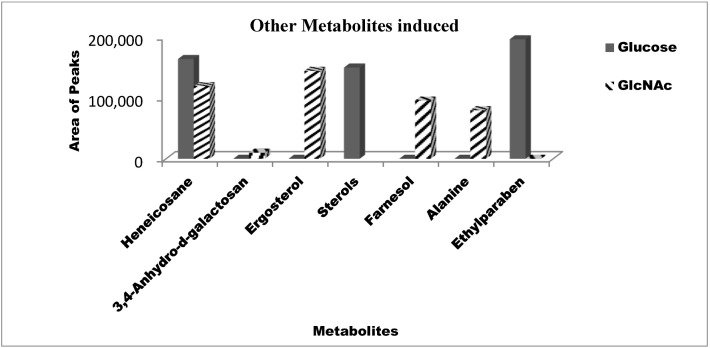
Comparison of different metabolites expressed under glucose- and GlcNAc-induced conditions. Black blocks represent the metabolites induced in glucose-grown cells, whereas striped blocks represent N-acetylglucosamine (GlcNAc)-induced cells. Here, the area of the peaks corresponds to the amount of metabolites incited in different carbon-induced cells.

To compare the low molecular weight compounds (metabolites) in yeast and hyphal cells, we also carried out metabolite profiling [32]. Using GC/MS, nontargeted metabolite profiling was carried out. Before GC–MS, we analysed the sample for HPLC to check the differential expression of metabolites in glucose- vs. GlcNAc-grown samples (Figure 5). Typically, the extracts of cells were fed glucose and GlcNAc resolved approximately 90 peaks. A high degree of confidence was used to identify 23 metabolites, which were then widely categorized (Table 1). A GC/MS study showed that during GlcNAc-induced morphogenesis, the majority (40%) of the detected metabolites were reduced, and approximately 20% showed increasing abundance seen in Figure 6. Most of the metabolites are present in both, such as ascorbic acid (interferes with the yeast-to-hypha transition in *C. albicans*) and arachidic acid (*C. albicans* produces extracellular prostaglandin(s), which have a critical function in the hyphal formation and host cell damage). Linoleic acid and several fatty acids, including conjugated linoleic acid (CLA), were later revealed to inhibit *Candida albicans* germ tubes. UDP-galactose affects cell wall integrity and morphology even in the absence of galactose. The accumulation of farnesol blocks the morphological shift from yeast to hyphal formation at high cell densities. Ethylparaben is an antifungal agent. Ergosterol, a sterol found on the cell membranes of fungi, works to preserve the integrity of cell membranes. A crucial biochemical mechanism in mitochondria that produces energy is the TCA cycle. On the other hand, the TCA cycle serves as a major metabolic mechanism that allows many substances, including amino acids, to be converted into intermediates that can then be used to synthesize specific amino acids. Acetyl CoA is formed due to glycolysis, glyoxylate, and dicarboxylate metabolism, and fatty acid metabolism. NADH is formed from ethanol fermentation, fatty acid, and fructose metabolism. These metabolites are found in glucose-induced cells, whereas these are utilized in GlcNAc-mediated cells. Flavin adenine dinucleotide is a metabolite for fatty acid metabolism, riboflavin metabolism, steroid biosynthesis, and the citric acid cycle which is found in the GlcNAc borne cells. The difference in the concentration of each metabolite has been shown in Figure 7. Therefore, from this experiment, it can be inferred that fatty acids are utilized by GlcNAc-induced cells for acetyl-CoA formation, which leads to the formation of energy (ATP), which is needed for the development of hyphae and extramembranous structures.

### 3.5. Minimum Inhibitory Concentration (MIC) Study

To understand the effect of AmpB on GlcNAc-grown Candida cells compared with glucose-grown Candida cells, we estimated the minimum inhibitory concentration (MIC). The minimum inhibitory concentration (MIC) can be defined as the AmpB concentration where there was a 50% drop in absorbance in comparison to the control absorbance [(Glucose = 1.1); (GlcNAc = 1)]. The MIC for the growth of glucose-induced *Candida albicans* cells is 6 µg/mL, whereas the MIC for the growth of GlcNAc-induced *Candida albicans* cells is 3 µg/mL, as shown in Table 2. Therefore, we can clearly see the difference in minimum inhibitory concentration, and it can be interpreted that GlcNAc-induced cells are more sensitive to AmpB as it forms hyphae, and the ergosterol present in those membranes might be more affected. The simplified model for AmpB’s biological action toward *C. albicans* cells is based on the illustrations that AmpB binds to the cell walls of mature cells; the antibiotic prevents budding-stage daughter cells from forming a functional cell wall; and the antibiotic is either found in ergosterol–AmpB bulk extramembranous structures or binds to the cell membrane and enters the cell [27,28].

## 4. Conclusions

GlcNAc is used as a nutrient and a signalling molecule by species ranging from bacteria to fungi to humans. It is a ubiquitous source of carbon in the ambient environment and within a mammalian host. The GlcNAc catabolic pathway of *C. albicans* allows it to use GlcNAc as a carbon source. GlcNAc serves as a signalling molecule that can cause filamentous development and a transformation from white to opaque in *C. albicans* as well as a source of nutrients. *C. albicans* is an excellent model system to further investigate the underlying molecular processes of GlcNAc sensing and use since these two morphological changes have been widely explored in this fungus. There are two ways that GlcNAc might cause signalling in *C. albicans*. The first is that it can function as a signalling molecule independently of catabolism, and the second is because catabolism can result in the extracellular environment becoming alkaline, which acts as a further inducement for hypha formation. Additionally, GlcNAc stimulates the translation of virulence genes in *C. albicans*, suggesting that it may have an impact on pathogenesis.

Here, we studied *Candida albicans* cells induced by different carbon sources and concluded that different carbon sources can change the morphology from yeast to hyphae and can also induce different metabolites that help the formation of hyphae and pathogenesis. Yeasts are linked to commensalism in mucocutaneous infection systems, such as oropharyngeal candidiasis, but filamentous forms (hyphae and pseudohyphae) are linked to tissue invasion and damage. Yeasts, pseudohyphae, and hyphae all appear to play a part in disseminated illness, such as abscesses within the host’s internal organs.

The majority of metabolites that aid the processes of oxidation, the glyoxylate cycle, the TCA cycle, and glucose synthesis are present in both glucose- and GlcNAc-induced cells. However, glucose-induced cells are where the majority of fatty acids are located. Therefore, it can be concluded from this experiment that GlcNAc-induced cells use fatty acids for the creation of acetylcoA, which results in the production of energy (ATP), which is required for the growth of hyphae and extramembranous structures.

The drug AmpB inhibits the budding stage daughter cells from developing a functional cell wall; it attaches to the cell walls of mature cells; it is either present in ergosterol-AmpB bulk extramembranous structures or adheres to the cell membrane and joins the cell. These examples form the basis of the simplified model for AmpB’s biological action toward *C. albicans* cells. Therefore, our results suggest that the MIC for the growth of glucose-induced *Candida albicans* cells is 6 µg/mL, whereas the MIC for the growth of GlcNAc-induced *Candida albicans* cells is 3 µg/mL. We can clearly see the difference in minimum inhibitory concentration, and it can be said that GlcNAc-induced cells are more prone to AmpB as it forms hyphae, and the ergosterol present in those membranes might be more affected. These will expand the pool of pharmacological targets for antivirulence drugs with distinctive modes of action.

## Figures and Tables

**Table 1:** Peak areas of metabolites expressed in glucose- and GlcNAc-induced cells.

Metabolites	Glucose	GlcNAc
Acetic acid	75,523	66,024
Oxalic acid	19,379	35,533
Formic acid	139,232	0
Carbamic acid	0	46,075
Carbonic acid	0	24,857
Malonic acid	225,082	183,445
Stearic acid	217,183	105,159
Trichloroacetic acid	177,869	77,711
Ascorbic acid	114,099	0
Pthalic acid	466,065	422,575
Erucic acid	272,754	0
Oleic Acid	336,842	0
Arachidic acid	138,511	0
Linoleic acid	183,765	0
Myristic acid	212,820	0
6-Aminocaproic acid	103,442	0
Heneicosane	163,744	119,053
3,4-Anhydro-d-galactosan	0	10,058
Ergosterol	0	144,365
Sterols	149,667	
Farnesol	0	95,425
3,4-Dihydroxyphenylglycol	2,158,059	0
Ethylparaben	196,123	0
Alanine	0	79,622

**Table 2:** Absorbance of different concentrations of AmpB used against *Candida albicans*.

Carbon	AmpB	100 µg/mL	50 µg/mL	25 µg/mL	12 µg/mL	6 µg/mL	3 µg/mL	1.5 µg/mL	0.7 µg/mL	0.3 µg/mL	0.1 µg/mL	YNB+ DMSO (WC) *	YNB (WC) *
Glucose		0.4430 ± 0.203	0.3765 ± 0.175	0.7372 ± 0.564	0.7737 ± 0.598	**0.6877 ± 0.237**	0.859 ± 0.454	0.9010 ± 0.264	1.2517 ± 0.061	0.7246 ± 0.295	0.5367 ± 0.108	0.0264 ± 0.042	0.0330 ± 0.039
GlcNAc		0.0646 ± 0.007	0.1367 ± 0.008	0.1952 ± 0.116	0.2663 ± 0.125	0.7277 ± 0.060	**0.4962 ± 0.111**	0.4242 ± 0.022	0.4723 ± 0.012	0.9434 ± 0.161	0.5266 ± 0.087	0.0283 ± 0.044	0.0339 ± 0.042

Different concentrations of AmpB ranging from 100 µg/mL to 0.1 µg/mL were used to check the MIC values for *Candida albicans* cells grown on two different carbon sources, glucose, and GlcNAc. Absorbance that was recorded through a microplate reader against different concentrations of AmpB was standardized (mean ± standard deviation). The MIC values have been marked with bold. (WC) * denotes without culture.

## Data Availability

Data are available on reasonable request from the corresponding author.
